# Faecal Bacterial and Short-Chain Fatty Acid Profiles in Response to 48 h FODMAP Intervention Prior to Endurance Exercise

**DOI:** 10.3390/nu18121886

**Published:** 2026-06-11

**Authors:** Rachel Scrivin, Isabel Martinez, Kayla Henningsen, Gary Slater, Rebekah Henry, Dovile Anderson, Ricardo J. S. Costa

**Affiliations:** 1 Sports and Health Faculty, Toi Ohomai Institute of Technology, Tauranga 3112, New Zealand; rachel.scrivin@toiohomai.ac.nz; 2 School of Health, University of the Sunshine Coast, Sippy Downs, QLD 4556, Australia; gslater@usc.edu.au; 3 Department of Nutrition Dietetics & Food, Faculty of Medicine, Nursing and Health Sciences, Monash University, Clayton, VIC 3168, Australia; isabel.martinez@monash.edu (I.M.); kayla.henningsen@monash.edu (K.H.); 4 School of Public Health and Preventive Medicine, Monash University, Clayton, VIC 3168, Australia; rebekah.henry@monash.edu; 5 Monash Proteomics and Metabolomics Platform, Monash Institute of Pharmaceutical Sciences, Parkville, VIC 3052, Australia; dovile.anderson@monash.edu

**Keywords:** acetate, butyrate, exercise-induced gastrointestinal syndrome, fermentable carbohydrates, gastrointestinal microbiome, gastrointestinal symptoms, propionate

## Abstract

**Background/Objectives**: Short-term low-fermentable oligo-, di-, and monosaccharide and polyol (FODMAP) diets can reduce exercise-associated gastrointestinal symptoms (Ex-GIS); however, their effects on the gut microbiome, short-chain fatty acids (SCFAs), and gastrointestinal biomarkers remain unclear. This study explored the effects of 48 h dietary FODMAP manipulation within a high-carbohydrate diet on faecal bacterial and SCFA profiles, and their relationships with exercise-induced gastrointestinal syndrome (EIGS) biomarkers, Ex-GIS, and performance. **Methods**: Twelve endurance athletes experiencing Ex-GIS were randomly allocated to a 48 h high-carbohydrate (mean ± SD: 12.1 ± 1.8 g∙d^−1^)–high-FODMAP (HC-HFOD) (54.8 ± 10.5 g∙d^−1^) and a 48 h high-carbohydrate–low-FODMAP (HC-LFOD) (3.0 ± 0.2 g∙d^−1^) diet before 2 h of running at 60% $\dot{V}$O_2max_, followed by a 1 h distance test (22.9 ± 1.2 °C, 46 ± 8% RH). Baseline faecal samples were collected before exercise trials to determine faecal bacterial and SCFA profiles. Blood samples were collected pre- and post-exercise to determine plasma I-FABP, sCD14, and CRP concentrations. Ex-GIS were recorded every 15 min throughout exercise. **Results**: Faecal bacterial α-diversity and relative abundance (RA%) at the phylum level were unchanged following both diets, while several family- and genus-level taxa RA% values were changed (*p* < 0.05), with greater shifts after HC-HFOD. HC-HFOD significantly increased faecal total-SCFA (*p* = 0.004), acetic (*p* = 0.002), and butyric (*p* = 0.028) acid concentrations. Strong positive and negative correlations between bacterial RA% and EIGS biomarkers and Ex-GIS were observed. Strong negative correlations with bacterial RA% and performance were observed. **Conclusions**: The 48 h HC-HFOD resulted in greater increases in bacterial RA% and SCFA concentrations compared with baseline. Bacterial RA% correlated bidirectionally with EIGS biomarkers and Ex-GIS, alongside strong negative associations with performance.

## 1. Introduction

Prolonged strenuous exercise has been shown to promote significant disturbances to gastrointestinal integrity and function [1]. Disturbances in the intestinal epithelial layer can increase intestinal permeability, leading to translocation of luminal originating pathogens (i.e., bacteria and bacterial endotoxins) into systemic circulation and subsequently prompting systemic immune responses [2,3]. In addition, disturbances to gastrointestinal function may lead to impaired gastrointestinal motility, digestive function and/or nutrient absorption [4]. The aetiology and pathophysiology of these disturbances to gastrointestinal integrity and function are well described in the literature, and termed exercise-induced gastrointestinal syndrome (EIGS) [1,5]. An outcome of EIGS is exercise-associated gastrointestinal symptoms (Ex-GIS), which may include abdominal bloating and/or pain, urge to defecate or regurgitate, and/or nausea during and/or after exercise [1,5]. These symptoms may be an inconvenience as a result of exercise per se, or may be an indication of clinical implications, such as gastroparesis, ileus, reversible colitis, sepsis, and/or systemic inflammatory response [6,7,8].

Some of the symptoms commonly reported by athletes during exercise are similar to those diagnosed with irritable bowel syndrome (IBS) [9]. IBS is a disorder of gut–brain interaction, characterised by recurrent abdominal pain related to a change in gastrointestinal function and bowel habit [10,11]. An effective dietary intervention to reduce gastrointestinal symptoms and improve quality of life for those with IBS is a low fermentable oligo-, di-, and monosaccharide and polyol (FODMAP) diet [9,12,13,14]. FODMAPs are highly osmotic and draw water into the gut, increasing luminal contents [9,11]. In addition, bacteria then rapidly ferment FODMAPs, producing gases [e.g., hydrogen (H_2_), methane (CH_4_), and carbon dioxide (CO_2_)]. An outcome of bacterial fermentation of FODMAPs is short-chain fatty acid (SCFA) production, which may further increase colonic contents [15,16]. An increase in colonic contents can alter gastrointestinal motility (e.g., ileal braking mechanisms) [4], which may lead to abdominal pain due to visceral hypersensitivity [17]. Given the overlap in gastrointestinal symptoms between those with IBS and athletes, it is not surprising that many athletes have also successfully implemented a low-FODMAP diet to reduce Ex-GIS [18,19,20]. Laboratory-controlled experimental studies have also shown that a 24–48 h low-FODMAP (<5 g∙d^−1^) diet before prolonged strenuous exercise (e.g., 2 h steady state running in temperate or hot ambient conditions with or without a 1 h performance test) reduces Ex-GIS severity [21,22].

The increase in Ex-GIS severity reported by athletes consuming high-FODMAP diets is likely due to the prebiotic nature of FODMAPs, where luminal microbial agents (i.e., bacteria) ferment FODMAPs, which produce SCFA [2]. The prebiotic effects of dietary FODMAPs have been associated with greater faecal bacterial α-diversity and relative abundance % (RA%) and/or of commensal SCFA-producing bacteria [23,24]. Acetate, butyrate, and propionate are the most abundant SCFAs in the large colon, which may protect intestinal epithelial integrity through their metabolism by colonocytes or exert disease-modulating effects [25]. The impact of commensal SCFA-producing bacteria could be beneficial in mitigating EIGS at the epithelial interface, such as mitigating epithelial disruption due to exertional stress and subsequent exercise-associated endotoxaemia and bacteraemia [2]. This impact has been investigated with a 24 h high-FODMAP (47 g∙d^−1^ total FODMAP) diet prior to exertional-heat stress (EHS), resulting in significantly lower intestinal epithelial injury [e.g., plasma intestinal fatty acid binding protein (I-FABP) concentration] and less disturbance of systemic bacterial endotoxin profile compared to a 24 h low-FODMAP (2 g∙d^−1^) diet [21]. A subsequent secondary analysis showed that plasma and faecal bacterial RA% varied between high- and low-FODMAP diets, and higher faecal and plasma SCFA concentrations were observed in the high-FODMAP diet [2]. These promising preliminary findings warrant further investigation into athlete-specific dietary practices (e.g., high-carbohydrate diets) and longer dietary FODMAP lead-in periods; the impact on faecal bacterial α-diversity, RA%, and faecal SCFA concentrations; and correlations with EIGS biomarkers, Ex-GIS, and performance.

Therefore, the aim of the current study was to explore the impact of a 48 h high-carbohydrate (12 g∙kg∙d^−1^)–high-FODMAP (55 g∙d^−1^) (HC-HFOD) diet and an HC–low-FODMAP (3 g∙d^−1^) (HC-LFOD) diet on faecal bacterial α-diversity and RA% and faecal SCFA concentrations. Based on previous research in exercise gastroenterology and dietary interventions, a secondary aim was that an HC-HFOD diet would result in stronger associations with faecal bacterial α-diversity and RA% and faecal SCFA concentrations, and EIGS biomarkers, Ex-GIS, and exercise performance. It was hypothesised that a greater RA% of faecal SCFA commensal bacteria would be negatively associated with lower biomarkers of EIGS [i.e., reduced intestinal epithelial injury, bacterial endotoxins, systemic inflammatory responses, and orocaecal transit time (OCTT)] [2], and reduced Ex-GIS severity [24], with a positive association to improved performance (i.e., greater distance completed) [26].

## 2. Materials and Methods

### 2.1. Participants

The current study presents an extended sample analysis based on previously published research, where experimental procedures are described in full [22]. The original methods were abridged for the current study. Twelve (*n* = 10 males and *n* = 2 females) non-heat-acclimatised trained endurance athletes [mean ± SD: age, 42.0 ± 5.2 years; body mass, 72.1 ± 13.7 kg; height, 1.74 ± 0.08 m; body fat, 20.0 ± 8.2%; and maximum oxygen uptake ($\dot{V}$O_2max_), 53.2 ± 8.6 mL∙kg∙min^−1^] with recurrent Ex-GIS volunteered to participate in the original study. The exclusion criteria for the current study were previously described [22]. Participants provided written informed consent prior to the original study commencement. The local ethics committee approved the original study (Monash University Human Research Ethics Committee approval number 28804), and this study was conducted in accordance with the 2013 Helsinki Declaration for Human Research Ethics. This study was registered with the Australian New Zealand Clinical Trials Registry (Trial identification number ACTRN12624000060549).

### 2.2. Experimental Procedures

Baseline preliminary measures were taken before the first experimental trial. Anthropometric measurements were recorded [e.g., height (Harpenden stadiometer, Holtain Ltd., United Kingdom), body mass (Seca 813 electronic scales, Seca Group, Hamburg, Germany), and body fat mass (Seca 515 MBCA, Seca Group, Hamburg, Germany)]. A baseline mid-flow faecal sample was collected in a sterile faecal collection container (SARSTEDT Australia Pty Ltd., Mawson Lakes, South Australia, Australia) and immediately stored at −80 °C. Participants provided a baseline food and activity diary of the previous two days that was checked for accuracy by a research dietitian. The baseline dietary analysis comprised (*n* = 12) the following: total energy, 3000 ± 595 kcals·d^−1^; carbohydrate, 329 ± 93 g·d^−1^; protein, 140 ± 36 g·d^−1^; fat, 114 ± 28 g·d^−1^; fibre, 35 ± 15 g·d^−1^; and total FODMAP, 21.7 ± 15.8 g·d^−1^ [22]. Participants then completed a continuous incremental running test to volitional exhaustion on a motorised treadmill (Pulsar 3p, h/p/cosmos, Munich, Germany) that was conducted in temperate ambient conditions. The experimental trial treadmill running speed was determined to be 60% of $\dot{V}$O_2max_ (31.9 ± 5.2 mL∙kg∙min^−1^) and 1% treadmill gradient, extrapolated from the $\dot{V}$O_2_–work rate relationship data and then verified (8.8 ± 1.2 km∙h^−1^).

**Figure 1:**
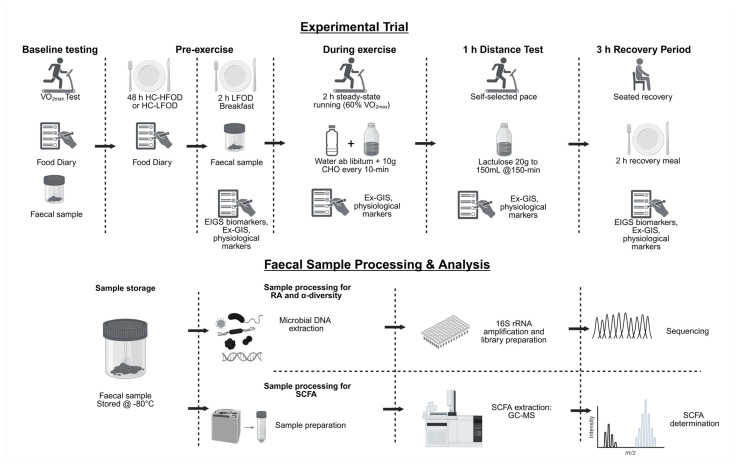
Schematic of the experimental trial and faecal sample processing plus analysis. Experimental trial procedures described in Scrivin et al. [22]. Faecal sample processing and analysis modified from Gaskell et al. [2]. CHO, carbohydrate; DNA, deoxyribonucleic acid; Ex-GIS, exercise-associated gastrointestinal symptoms; EIGS, exercise-induced gastrointestinal syndrome; GC-MS, gas chromatography–mass spectrometry; HC-HFOD, high-carbohydrate–high-FODMAP; HC-LFOD, high-carbohydrate–low-FODMAP; PCR, polymerase chain reaction; RA, relative abundance; SCFAs, short-chain fatty acids. Created in BioRender. Scrivin, R. (2026). https://BioRender.com/jbhvni7 (accessed on 4 June 2026).

The experimental procedures previously reported by Scrivin et al. [22] are illustrated schematically, with further faecal sample analytical methods described in Figure 1. A double-blind randomised crossover design was used for the current study. Participants were prescribed a 48 h HC-HFOD (energy, 4561 ± 406 kcal∙d^−1^; carbohydrate, 813 ± 101 g∙d^−1^; protein, 157 ± 11 g∙d^−1^; fat, 68 ± 8 g∙d^−1^; fibre, 45 ± 1 g∙d^−1^; and total FODMAP, 54.8 ± 10.5 g∙d^−1^) before one exercise trial, and an HC-LFOD (energy, 4877 ± 348 kcal∙d^−1^; carbohydrate, 888 ± 71 g∙d^−1^; protein, 140 ± 12 g∙d^−1^; fat, 78 ± 9 g∙d^−1^; fibre, 53 ± 1 g∙d^−1^; and total FODMAP, 3.0 ± 0.2 g∙d^−1^) diet before the other exercise trial. The specific FODMAP breakdown for HC-HFOD was excess fructose (3.5 g), lactose (46.8 g), fructo-oligosaccharide (4.0 g), galacto-oligosaccharide (2.3 g), and total polyols (1.2 g); and HC-LFOD was excess fructose (0.5 g), lactose (0.0 g), fructo-oligosaccharide (1.8 g), galacto-oligosaccharide (0.6 g), and total polyols (0.1 g). A 7-day washout period separated the trials, during which participants consumed their habitual diet, followed by the subsequent 48 h dietary intervention. On the day of the exercise trial, participants consumed a standardised low-FODMAP breakfast (energy, 924 kcal; carbohydrate, 138 g; protein, 37 g; fat, 23 g; and total FODMAP, 0.5 g). Dietary control processes, including blinding and compliance, were reported previously in Scrivin et al. [22].

### 2.3. Sample Processing and Analysis

As previously described by Scrivin et al. [22], breath samples (20 mL) were analysed in duplicate (CV%: HC-HFOD = 3.4%; HC-LFOD = 1.8%) for H_2_ content using a gas-sensitive analyser (BreathTracker Digital Microlyzer, Quintron, Milwaukee, WI, USA). To estimate changes in plasma volume relative to baseline and to correct plasma variables, whole-blood haemoglobin and haematocrit values were measured. Haemoglobin (HemoCue Hb 201^+^, HemoCue AB, Angelholm, Sweden) (CV%: HC-HFOD = 1.7%, HC-LFOD = 1.4%) was determined using heparin whole blood. Haematocrit was measured in triplicate (CV%: HC-HFOD = 1.1%; HC-LFOD = 0.9%) using micro-haematocrit tubes centrifuged for 2 min at 10,000 rpm. The remaining heparin and EDTA blood samples were centrifuged within 15 min of sample collection at 4000 rpm (1500× *g*) for 10 min at 4 °C. Plasma was aliquoted into 1.7 mL microtubes and frozen at −80 °C until analysis. Plasma concentrations of intestinal fatty acid binding protein (I-FABP) (HK406, Hycult Biotech, Uden, The Netherlands), soluble cluster of differentiation 14 (sCD14) (HK320, Hycult Biotech, Uden, The Netherlands), and C-reactive protein (CRP) (RayBio Human CRP, Ray Biotech, Norcross, GA, USA) were determined by ELISA. Samples were analysed in duplicate on the same day, as per manufacturer instructions, with standards and controls on each plate, and each participant assayed on the same plate. The CV% for plasma I-FABP, sCD14, and CRP concentration variables was 5.0%, 3.8%, and 3.1%, respectively.

### 2.4. Faecal Sample Bacterial Profiling and SCFA Determination

Applying the same methods described by Henningsen et al. [3], faecal samples were thawed at room temperature and homogenised, and then 0.2–0.3 g of each sample was transferred to a 2 mL dry garnet-bead microtube before the addition of a bead solution. Cell lysis, sample purification, and DNA extraction were performed as per the manufacturer’s instructions (PowerFecal DNA isolation kit, Qiagen, Germantown, TN, USA). Blank control samples, using pyrogen/DNAse/RNAse-free water, were run simultaneously, in duplicate. Purified extracted DNA (50 µL sample) was immediately frozen at −20 °C prior to bacterial gene sequencing. The extracted genomic DNA was sent to Monash Genomics and Bioinformatic Platform (Monash University, Clayton, Australia) for targeted PCR amplification of the V3–V4 region of the 16S rRNA gene and sequencing.

Applying the methods described by Gaskell et al. [2], phylum-, family-, and genus-level amplicon sequence variant (ASV) counts were calculated by dividing the number of reads for each taxon by the number of reads in the faecal samples, taking into account background counts detected in blank control samples. 16S rRNA sequences per faecal sample ranged from 138,805 to 265,021. To avoid the risk of including artefact values in data analysis resulting from potential contamination during sample handling (e.g., sample collection, processing, and analysis procedures), for ASV, bacterial groups with ≥0.5% RA, respective to the determination medium, were included for data analysis. Bacterial calculations of phyla (*n* = 4), family (*n* = 20), and genus (*n* = 35) taxa were adequately detected for α-diversity [i.e., Shannon Equitability Index (SEI)] and RA% (≥0.5%).

Faecal samples were sent to the Monash Proteomics and Metabolomics Platform for total and differential (e.g., acetic acid, butanoic acid, and propionic acid) SCFA determination. Faecal samples were extracted with 60 uL·mg^−1^ dry weight of methanol (MeOH), and a portion of the extract was derivatised using a mixture of 3-NPH HCl (3-nitrophenylhydrazine hydrochloride) (Sigma-Aldrich, St. Louis, MO, USA) and EDC·HCl (*N*-(3-dimethylaminopropyl)-*N*′-ethylcarbodiimide hydrochloride) (Sigma-Aldrich, St. Louis, MO, USA) in 7.5% pyridine 50% acetonitrile, as per adapted published procedure [27]. Calibrants and spiked quality control (QC) samples were derivatised alongside this study’s samples. Internal standards (ISTDs) for all analytes were prepared by derivatising a mixed solution of SCFA using ^13^C_6_-3-NPH·HCl (3-nitrophenyl-1,2,3,4,5,6^−13^C_6_)hydrazine hydrochloride (Cayman Chemical, Ann Abor, MI, USA) and were added to all samples, calibrants, and QC samples. Liquid chromatography–mass spectrometry (LC-MS) analysis was performed using a Q-Exactive mass spectrometer (Thermo Fisher Scientific, Waltham, MA, USA) coupled to a Dionex Ultimate 3000 RSLC chromatography system (Thermo Fisher Scientific, Waltham, MA, USA) employing a Zorbax RRHD Eclipse Plus C_18_ column (1.8 µm particle size, 2.1 × 100 mm) (Agilent Technologies, Santa Clara, CA, USA). Data were processed using the TraceFinder 4.1 software (Thermo Fisher Scientific, Waltham, MA, USA). Accuracy of calibrants and spiked QC samples was within the recommended ±15% difference; calibrant R^2^ < 0.98.

### 2.5. Statistical Analysis

Confirmation of statistical power for the primary research was previously described [22]. Descriptive data are presented as mean ± SD, and primary and secondary variables as mean and 95% confidence intervals (CI), as indicated. Data in figures are presented as mean ± SEM for clarity, where indicated. Prior to comparative statistical tests, the Shapiro–Wilks test of normality was performed. Paired-samples *t*-tests or Wilcoxon tests were used to compare baseline, HC-HFOD, and HC-LFOD diets. For comparisons among multiple group datasets, one-way ANOVA with Tukey’s post hoc tests was used to adjust for multiple pairwise comparisons within each analysis. Pearson’s or Spearman’s rank correlation coefficients, where appropriate, were utilised to determine the strength of the linear relationship between faecal bacterial and SCFA profiles with gastrointestinal integrity biomarkers, OCTT, Ex-GIS, and exercise performance. The strength of the linear relationship between the variables was determined as very weak, at <0.200; weak, at 0.200–0.399; moderate, at 0.400–0.599; and strong, at ≥0.600. In addition, Cohen’s standardised measurement of effect size was applied where appropriate for small (*d* = 0.20), medium (*d* = 0.50), and large (*d* = 0.80) effects [28]. Given the exploratory nature of this study and the number of outcomes assessed, no further adjustments for multiplicity across analyses were applied; therefore, *p*-values were interpreted descriptively. Statistical analyses were performed using SPSS (V.29.0, Chicago, IL, USA), with significance accepted at *p* ≤ 0.05.

## 3. Results

### 3.1. EIGS Biomarkers, Ex-GIS, and Exercise Performance

A full description of EIGS biomarkers, Ex-GIS, and exercise performance results are reported in Scrivin et al. [22]. Pre- to peak post-exercise changes (mean and 95% CI) for plasma I-FABP [HC-LFOD 1120 (749, 1508) pg·mL^−1^ and HC-HFOD 1148 (709, 1607) pg·mL^−1^], sCD14 [HC-LFOD 2396 (2064, 2742) ng·mL^−1^ and HC-HFOD 2562 (2085, 3060) ng·mL^−1^], and CRP [HC-LFOD 6.0 (4.0, 8.1) ug·mL^−1^ and HC-HFOD 5.96 (3.5, 8.6) ug·mL^−1^] concentrations were observed. The breath H_2_ turning point for HC-LFOD 95 (64, 126) min and HC-HFOD 83 (50, 115) min was observed. There was no statistical difference in EIGS biomarkers between HC-LFOD and HC-HFOD.

The incidence of total Ex-GIS pre-exercise, during exercise, and in the recovery period was 25% (*n* =3), 92% (*n* = 11), and 58% (*n* = 7) for HC-LFOD; and 58% (*n* = 7), 100% (*n* = 12), and 58% (*n* = 7) for HC-HFOD, respectively. Significantly higher Ex-GIS incidence was observed pre-exercise (*p* = 0.046, *d* = 0.58) and in the recovery period (*p* = 0.041, *d* = 0.59) on HC-HFOD, compared to HC-LFOD.

The severity of total Ex-GIS (summative accumulation and range) pre-exercise, during exercise, and in the recovery period was [15 (2, 10)], [220 (3, 75)], and [198 (2, 72)] for HC-LFOD; and [52 (3, 11)], [314 (2, 49)], and [60 (1, 20)] for HC-HFOD, respectively. Significantly greater total Ex-GIS severity was observed pre-exercise (*p* = 0.042, *d* = 0.69) and in the recovery period (*p* = 0.043, *d* = 0.69) on HC-HFOD compared to HC-LFOD.

The total distance completed (mean and 95% CI) during the 1 h distance performance test for HC-LFOD and HC-HFOD was 10.0 (9.1, 10.9) km and 9.9 (8.9, 10.9) km, respectively. There was no statistical difference between HC-LFOD and HC-HFOD.

### 3.2. Faecal Bacterial Taxa

The faecal bacterial taxa α-diversity (SEI) and predominant phyla, family, and genus bacterial groups are highlighted in Table 1. The identification of RA% of bacterial phyla groups in faecal samples included *Firmicutes*, *Bacteroidota*, *Actinobacteriota*, and *Proteobacteria*. Baseline, HC-LFOD, and HC-HFOD faecal bacterial phyla SEI did not differ significantly [*x*^2^(2) = 1.225, *p* = 0.542]. Baseline RA% of predominant bacterial family groups in faecal samples in which the cohort presented >2% included *Lachnospiraceae*, *Ruminococcaceae*, *Bacteroidaceae*, *Prevotellaceae*, *Bifidobacteriaceae*, *Selenomonadaceae*, and *Erysipelatoclostridiaceae.* Baseline, HC-LFOD, and HC-HFOD faecal bacterial family SEI did not differ significantly [*F*(2,33) = 0.166, *p* = 0.847]. Baseline RA% of predominant bacterial genus groups in faecal samples, in which the cohort presented >2%, included *Bacteroides*, *Faecalibacterium*, *Blautia*, *Prevotella*, *Agathobacter*, *Subdoligranulum*, *Bifidobacterium*, *Hallii*, *Ruminococcus*, *Fusicatenibacter*, *Coprococcus*, and *Megamonas.* Baseline, HC-LFOD, and HC-HFOD faecal bacterial genus SEI did not differ significantly [*F*(2,33) = 0.251, *p* = 0.779].

**Figure 2:**
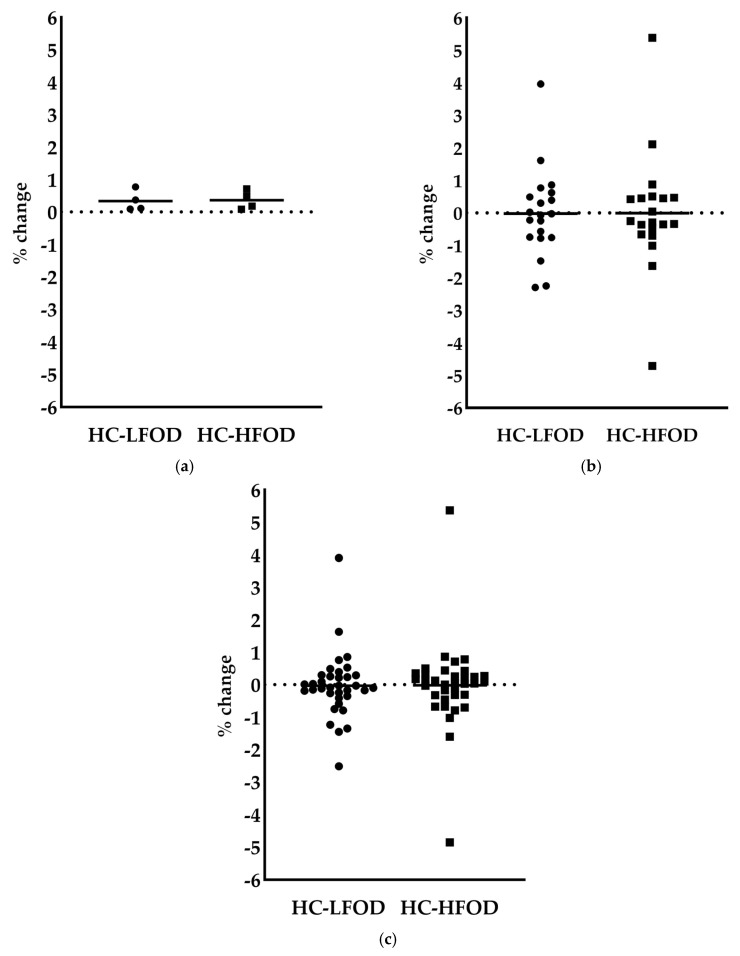
Overall relative change (%) in faecal bacterial relative abundance from baseline compared to high-carbohydrate–low-FODMAP (HC-LFOD) or high-carbohydrate–high-FODMAP (HC-HFOD) interventions at (**a**) phylum, (**b**) family, and (**c**) genus taxonomic levels. FODMAP, fermentable oligo-, di-, and monosaccharide and polyol; (**―**) mean.

At the phylum, family, and genus taxonomic levels, no significant differences in relative change between baseline and HC-LFOD or HC-HFOD were observed for faecal bacterial SEI. At the phylum taxonomic level, no significant differences in relative changes between baseline and HC-LFOD or HC-HFOD were observed for faecal bacterial RA (Table 2 and Figure 2a). At the family and genus taxonomic levels, significant differences in relative changes between baseline and HC-LFOD and HC-HFOD (Table 2) were observed in faecal bacterial RA, with greater differences observed between baseline and HC-HFOD (Figure 2b,c).

### 3.3. Faecal Bacterial Relative Abundance and Correlations to EIGS, Ex-GIS, and Performance

No correlations were observed between SEI and pre- to peak post-exercise magnitude of change for plasma I-FABP, sCD14, or CRP concentrations and Ex-GIS. A moderate negative correlation was observed between SEI and OCTT (Table 3). Several positive and negative correlations between faecal bacterial RA% and EIGS biomarkers, as well as Ex-GIS, were observed across different bacterial groups at the phylum, family, and genus taxonomic levels (Table 3). Several strong negative correlations were observed at all taxonomic levels between faecal bacterial RA% and performance.

### 3.4. Faecal Short-Chain Fatty Acid Concentrations

**Figure 3:**
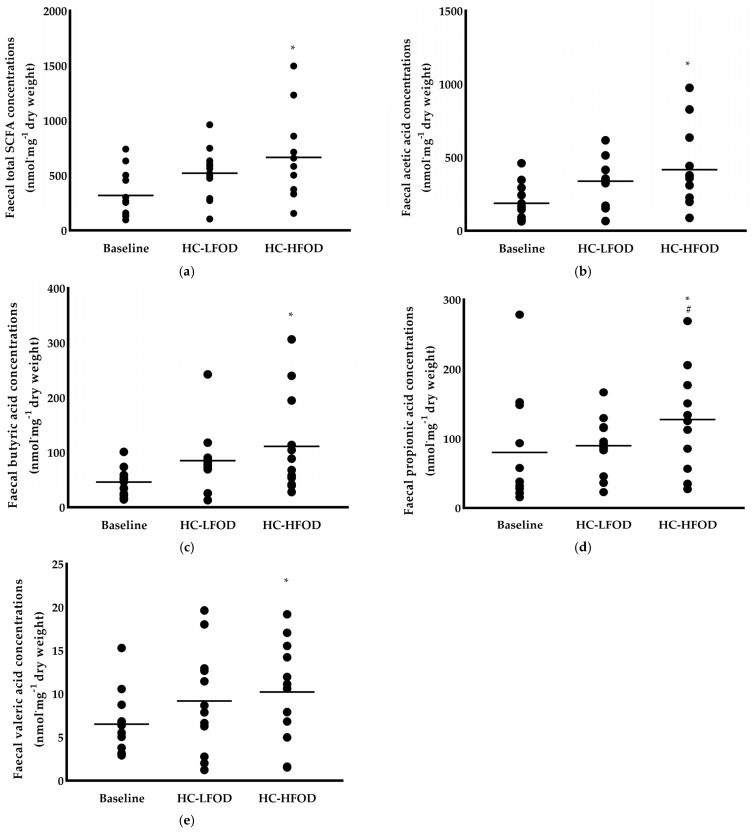
Resting faecal total-SCFA (**a**), acetic acid (**b**), butyric acid (**c**), propionic acid (**d**), and valeric acid (**e**) concentrations at baseline, high-carbohydrate–low-FODMAP (HC-LFOD), and high-carbohydrate–high-FODMAP (HC-HFOD) diets. FODMAP, fermentable oligo-, di-, and monosaccharide and polyol. Mean (**―**) and individual responses (●) (*n* = 12). * Baseline and HC-HFOD difference (*p* < 0.05). ^#^ HC-LFOD and HC-HFOD difference (*p* < 0.05).

Significant main effects were found for faecal total-SCFA [*F*(2,33) = 4.44, *p* = 0.020], acetic acid [*x*^2^(2) = 8.711, *p* = 0.013], and butyric acid [*x*^2^(2) = 7.409, *p* = 0.025] concentrations. No significant differences in faecal total- or differential-SCFA concentrations were found between baseline and HC-LFOD. However, significantly higher resting faecal total-SCFA (*p* = 0.014, *d* = 0.84 Figure 3a), acetic acid (*p* = 0.013, *d* = 1.02 Figure 3b), butanoic acid (*p* = 0.025, *d* = 0.73 Figure 3c), propionic acid (*p* = 0.033, *d* = 0.71 Figure 3d), and valeric acid (*p* = 0.023, *d* = 0.76 Figure 3e) concentrations were found on HC-HFOD compared to baseline. In addition, faecal propionic acid (*p* = 0.033, *d* = 0.71 Figure 3d) concentrations was significantly greater on HC-HFOD compared with HC-LFOD.

### 3.5. Faecal Short-Chain Fatty Acid Concentrations and Correlations to EIGS, Ex-GIS, and Performance

A moderate correlation was observed between faecal propionic acid with plasma sCD14 concentration (*r_s_* = −0.422, *p* = 0.040). No other correlations were found between faecal total or differential SCFA concentrations and EIGS biomarkers, Ex-GIS, or performance.

## 4. Discussion

The aim of this study was to explore the impact of 48 h high-carbohydrate–high-FODMAP and high-carbohydrate–low-FODMAP diets on faecal bacteria α-diversity, RA%, and faecal SCFA concentrations. In addition, this study aimed to explore relationships between faecal bacterial profiles and EIGS biomarkers, Ex-GIS, and exercise performance. In accordance with the hypothesis, compared to baseline, HC-HFOD resulted in greater changes in faecal bacterial groups (i.e., family and genus taxa), and total and differential faecal SCFA concentrations than HC-LFOD. When reviewing correlations between faecal bacterial groups and EIGS biomarkers and Ex-GIS, several moderate-to-strong positive and negative correlations were observed, but not with faecal SCFA profiles. Contrary to the hypothesis, several strong negative correlations were observed between faecal bacterial phyla, family, and genus, and exercise performance. HC-HFOD increased commensal bacteria and faecal SCFA concentrations that are potentially beneficial for mitigating EIGS, but at the expense of increased Ex-GIS severity. For the HC-HFOD, there were more positive correlations between bacterial groups and Ex-GIS, suggesting that increasing RA% (including commensal bacterial groups) may exacerbate Ex-GIS. All significant correlations between exercise performance and RA% were negative; however, as these findings are exploratory, they should be interpreted with caution. This pattern may suggest that changes in commensal bacteria do not align with current assumptions that modulation of the gut microbiome necessarily improves performance [29,30,31,32].

### 4.1. Changes in α-Diversity and Absolute Relative Abundance of Bacteria

Investigations into the impact of the gut microbiome have gained interest across many sporting fields (e.g., elite race walking [33], running [3,34], cycling [35,36], and rugby [37]), with the suggestion that changes in faecal bacterial profiles and SCFA may have implications for gastrointestinal status and performance outcomes. In the current study, no differences in faecal bacterial α-diversity (i.e., SEI) were observed at any taxonomic level between HC-HFOD and HC-LFOD. These findings were similar to other research with endurance athletes, where either a dietary and/or exercise intervention does not appear to impact SEI. However, the RA% of bacteria at various taxonomic levels may change [2,3,38,39]. Gaskell et al. [26] conducted a review on whether nutritional supplements, and dietary and exercise interventions influence gastrointestinal microbiota and subsequent exercise performance. Eighteen studies were included in the review. Findings from the review support the current study, in which faecal bacterial α-diversity appears to remain stable across taxonomic groups, and is not easily influenced by short-term probiotic, dietary, or exercise interventions in active adults. Additionally, there was no reported influence on performance outcomes [26]. It is worth noting that the review by Gaskell et al. [26] highlighted the predominant inclusion of male participants across studies. Similarly, the current study included mainly males (i.e., 10 males and 2 females). It is possible that sex-related factors may influence microbial composition. It is also possible that despite standardising exercise intensity at 60% of $\dot{V}$O_2max_ to ensure comparable relative workload across participants, individual physiological responses may still have influenced microbial composition. Due to the small sample size, sex-specific and fitness-level analyses were not feasible. This limitation is acknowledged, and future research should include larger, more balanced cohorts.

In the research reported here, the predominant baseline phyla (i.e., *Firmicutes*, 67%; *Bacteroidota*, 27%; *Actinobacteriota*, 5%; and *Proteobacteria*, 0.7%), family (i.e., *Lachnospiraceae*, 32%; *Ruminococcaceae*, 19%; *Bacteroidaceae*, 16%; *Prevotellaceae*, 6%; *Bifidobacteriaceae*, 2.7%; *Selenomonadaceae*, 2.2%; and *Erysipelatoclostridiaceae*, 2.1%), and genus (i.e., *Bacteroides*, 16%; *Faecalibacterium*, 11%; *Blautia*, 8%; *Prevotella*, 6%; *Agathobacter*, 4%; *Subdoligranulum*, 3.4%; *Bifidobacterium*, 2.7%; *Hallii*, 2.7%; *Ruminococcus*, 2.6%; *Fusicatenibacter*, 2.5%; *Coprococcus*, 2.3%; and *Megamonas*, 2.2%) were identified. These findings were similar to previous gut microbiota data reported in other studies in endurance-trained athletes, with the same predominant bacteria observed at the phyla (i.e., *Firmicutes* and *Bacteroidota*) and family (i.e., *Lachnospiraceae* and *Ruminococcaceae*) levels [2,3,24,38,39]. Greater differences in predominant bacterial groups were evident at the genus level, most likely due to large individual variation in gut microbiota despite standardised experimental control [1]. While *Bacteroides*, *Faecalibacterium*, and/or *Blautia* were commonly reported among the three most abundant genera, studies also reported *Alistipes* [24], *Agathobacter* [38], *Prevotella* [39], *Roseburia*, and *Escherichia-Shigella* [2]. In addition to individual variation in gut microbiota, it is possible that other methodological factors contributed to variations in predominant bacteria observed at the genus level, including dietary interventions (e.g., dietary FODMAP manipulation and prebiotics) [2,22,24,38,39], dietary lead-in periods (e.g., 24 h, 48 h, and 8 weeks) [2,24,38,39], baseline or habitual dietary intake [3], and/or exercise protocols (e.g., EHS and ultramarathon) [2,3,38]. Furthermore, variability may arise from faecal sample collection, storage, and processing, as well as RNA extraction, sequencing, data processing, and bacterial taxa determination.

In this research, compared to baseline, a 48 h HC-HFOD resulted in greater and more substantial changes in bacterial RA% than HC-LFOD. Within the *Bacteroidota* phyla, changes were observed at the family and genus level for commensal bacterial groups [i.e., *Bacteroidaceae* family (+5.4%) and *Bacteroides* genus (+5.4%); *Barnesiellaceae* family (+0.5%) and *Barnesiella* genus (+0.4%); *Rikenellaceae* family (+0.9%) and *Alistipes* genus (+0.9%); and *Tannerellaceae* family (0.5%) and *Parabacteroides* genus (+0.5)]. A similar change for commensal bacteria was observed in HC-LFOD family and genus levels compared to baseline [i.e., *Barnesiellaceae* family (+0.6%) and *Barnesiella* genus (+0.6%); and *Rikenellaceae* family (+0.9%) and *Alistipes* genus (+0.8%)], which is likely due to the high carbohydrate load (i.e., 12 g·d^−1^) in both dietary interventions, irrespective of FODMAP load. However, HC-HFOD produced substantial shifts in both *Bacteroidaceae* family and *Bacteroides* genus, which was not observed in HC-LFOD, possibly due to significant FODMAP dietary load differences [i.e., HC-HFOD 55 g·d^−1^ vs. HC-LFOD 3 g·d^−1^) [22] promoting greater changes to gut microbiota [2,15]. Pathogenic bacterial groups (e.g., opportunistic pathogens) were lower in HC-HFOD [i.e., *Lachnoclostridium* genus (−0.7%); *gaurveauii* group of *Ruminococcus* genus (−0.3%); and *Prevotellaceae* family (−4.7%) and *Prevotella* genus (−4.8%)]. The reduction in these pathogenic bacteria was not significant in HC-LFOD. Taken together, HC-HFOD appears to drive greater changes in faecal RA% than HC-LFOD, with beneficial shifts toward commensal bacteria and concomitant reductions in opportunistic bacteria. These alterations may influence EIGS outcomes, Ex-GIS, and performance [26].

### 4.2. Faecal Bacterial and Correlations to EIGS, Ex-GIS, and Performance

The role of specific commensal or pathogenic bacteria likely to impact EIGS outcomes, Ex-GIS, and/or exercise performance was explored. In the present study, strong positive and negative correlations were observed with peak plasma I-FABP [e.g., *Bacteroidaceae* family (*r* = 0.637, *p* < 0.001) and *Rikenellaceae* family (*r* = 0.602, *p* = 0.002); *Bacteroides* genus (*r* = 0.661, *p* < 0.001), *Alistipes* genus (*r* = 0.602, *p* = 0.002), and *Megamonas* genus (*r* = −0.607, *p* = 0.002)], and CRP [e.g., *gaurveauii* group of *Ruminococcus* genus (*r* = −0.656 *p* = 0.002)] concentrations, and Ex-GIS [e.g., *Rikenellaceae* family (*r* = 0.603, *p* = 0.002), *Alistipes* genus (*r* = 0.603 *p* = 0.002), and *Dorea* (genus) (*r* = −0.614, *p* = 0.001)]. These findings are similar to other studies, which report both strong negative and positive correlations to various EIGS biomarkers and Ex-GIS. Bennett et al. [24] investigated faecal bacterial profiles and associations with EIGS biomarkers and Ex-GIS in response to EHS. Commensal and pathogenic bacterial groups were moderately correlated with greater plasma I-FABP concentrations [e.g., *Tenericutes* phyla (*r* = 0.446, *p* = 0.049) and *Verrucomicrobia* phyla *(r* = 0.450, *p* = 0.046); *Akkermansiaceae* family (*r* = 0.486, *p* = 0.030) and *Ruminococcaceae* family *(r* = 0.449, *p* = 0.047); and *Akkermansia* genus (*r* = 0.486, *p* = 0.030), *Ruminiclostridium-6* genus *(r* = 0.544, *p* = 0.001), and *Ruminiclostridium-9* genus *(r* = 0.457 *p* = 0.043)]. Meanwhile, positive and negative correlations were observed with systemic inflammatory profile [e.g., *Faecalibacterium* genus *(r* = 0.668, *p* = 0.009) and *Ruminiclostridium-9* genus (*r* = −0.557, *p* = 0.031)] and gastrointestinal symptoms [e.g., *Actinomycetaceae* family (*r* = 0.624, *p* = 0.003), *Prevotellaceae* family (*r* = −0.458, *p* = 0.042), *Defluviitaleaceae* family (*r* = −0.467, *p* = 0.038), *Prevotella-7* genus (*r* = −0.450, *p* = 0.047), *Rikenellaceae-RC9* genus (*r* = −0.490, *p* = 0.028), and *Desulfovibrio* genus (*r* = −0.470, *p* = 0.036)]. Similar findings were observed in an ultramarathon (80 km trail running event) where strong positive and negative correlations were observed with certain bacteria and Ex-GIS [i.e., *Proteobacteria* phyla (*r* = 0.838, *p* < 0.001) and *Firmicutes* phyla (*r* = −0.606, *p* = 0.028) were associated with greater total-GIS] [3]. Bennett et al. [24] performed a subgroup analysis of their EHS study and observed greater faecal bacterial α-diversity and RA%, between biological sex, age, and fitness status (i.e., females vs. males; younger vs. older athletes; and lower vs. higher fitness status). Evidence from Bennett et al. [24] demonstrates that individual athlete characteristics may meaningfully influence baseline gut microbiota composition and SEI and should be considered when analysing microbiota, EIGS, Ex-GIS, and performance outcomes. A subgroup analysis on SEI and RA% was not conducted in the current study, which is a limitation of the analysis. It is possible that individual athlete characteristics also impact the SEI stability and RA% of bacterial groups.

From an exercise-performance perspective, a novel finding from this study was the absence of any association between SEI and performance. Contrary to this study’s hypothesis, all significant associations between faecal bacterial RA% and performance were strongly negative [e.g., *Coriobacteriaceae* family (*r* = −0.647, *p* < 0.001), *Collinsella* genus (*r* = −0.638, *p* < 0.001), *Agathobacter* genus (*r* = −0.751, *p* < 0.001), and *Fusicatenibacter* genus (*r* = −0.654, *p* < 0.001], indicating that higher RA% of these bacterial groups was associated with a shorter distance covered in the performance test. This finding supports a previous review of gut microbiota and exercise studies, which found that faecal bacterial α-diversity (SEI) has little impact on performance; that RA% associations with performance were inconsistent across studies; and that any changes observed are most likely attributable to diet, fitness status, and study design [26]. Collectively, these findings do not fully support the commonly held view that altering the gut microbiome enhances performance, and they should be interpreted with caution. The results suggest that an HC-HFOD diet may protect against EIGS and Ex-GIS, potentially at the expense of exercise performance. While the underlying mechanisms remain unclear, plausible contributors include increased fermentation and SCFA production, leading to greater osmotic load, carbohydrate malabsorption, and exacerbation of Ex-GIS, all of which could impair performance. Additionally, individual variability in microbiome composition and tolerance to FODMAPs may have influenced these findings. It is also important to note that changes in microbial profiles were diet-induced, and any changes elicited by exercise were not assessed in the present study.

We acknowledge that the small sample size of the current study may limit the generalisability of these findings to broader athletic populations. In addition, given the relatively small sample size and large number of comparisons performed, there is an increased risk of both false-positive and false-negative findings. As such, these results should be interpreted as exploratory and require validation in larger, appropriately powered studies with correction for multiple testing. However, these novel findings may inform future research with larger, more diverse cohorts.

### 4.3. Faecal Short-Chain Fatty Acid Concentrations

To maintain gut health, it is beneficial to have a diverse, symbiotic gut microbiome with a high RA% of commensal SCFA-producing bacteria [40]. The predominant SCFAs in faecal samples are acetic, butyric, and propionic acids, which have been shown to enhance luminal immunity (e.g., stimulating antimicrobial protein secretion and activating innate immune responses) and strengthen the intestinal epithelial barrier (e.g., enterocyte proliferation, increased tight junction expression, and mucus production) [41]. These mechanisms inhibit the growth and adhesion of opportunistic pathogenic bacteria to the intestinal epithelial barrier [42]. Therefore, diets that have the potential to increase intestinal SCFA concentration (e.g., high-FODMAP diets) could offer gastrointestinal benefits for athletes undertaking prolonged strenuous exercise, given the observed changes in intestinal epithelial integrity and immune responses during exercise [2,21,22]. However, this relevance may be limited to the circulatory–gastrointestinal pathway of EIGS, which is typically asymptomatic but can have clinically significant implications (e.g., sepsis and systemic inflammatory response syndrome (SIRS)). Accordingly, SCFA-related benefits may be most relevant for athletes at increased risk of compromised gastrointestinal integrity, such as those participating in ultramarathon events and/or exercising in hot ambient environments [3,38].

In an EHS trial, a high-FODMAP (47 g∙d^−1^) intervention was associated with greater total faecal SCFA production, including acetic, butyric, and propionic acid concentrations, compared to low-FODMAP (2 g∙d^−1^) [2]. Lower EHS-induced intestinal epithelial injury (i.e., lower plasma I-FABP concentrations) was observed on high-FODMAP, suggesting that greater faecal SCFA concentrations may have provided some protective mechanism against EIGS. However, this may have been at the expense of greater reported Ex-GIS on high-FODMAP, highlighting a potential trade-off between gut health-related benefits and increased Ex-GIS [21]. In contrast, Rauch et al. [39] found no differences in faecal SCFA concentrations between an 8-week prebiotic supplementation and a placebo prior to EHS. This finding was attributed to large variation within and between participants, likely associated with a lower fermentable nutrient exposure [e.g., 16 g daily prebiotic formulation (i.e., excess fructose, lactose, and total polyols (0 g); fructo-oligosaccharide (0.4 g); galacto-oligosaccharide (3.7 g); and dietary fibre and resistant starch (not quantified)] vs. a 47 g daily FODMAP reported in Gaskell et al. [i.e., excess fructose (8.7 g); lactose (21.2 g); fructo-oligosaccharide (10.1 g); galacto-oligosaccharide (0.7 g); and total polyols (6.2 g)] [21]. Rauch et al. [39] also reported no differences in Ex-GIS. Findings in the current study are similar to those reported by Gaskell et al. [2], where greater total faecal SCFA levels, including acetic, butyric, propionic, and valeric acid concentrations, were observed on HC-HFOD (55 g∙d^−1^) compared to baseline. However, no differences in faecal SCFA concentrations were observed between baseline and HC-LFOD (3 g∙d^−1^). Only a moderate correlation with faecal propionic acid and plasma sCD14 concentrations (*r* = −0.413, *p* = 0.045) was observed. No other associations between faecal SCFA concentrations and EIGS, Ex-GIS, or performance were observed. As several correlations were observed between faecal bacterial RA% and EIGS, Ex-GIS, and performance, it is likely that the bacterial RA% had a greater impact than faecal SCFA concentrations in the current study.

## 5. Conclusions

HC-HFOD diets promoted greater changes in faecal bacterial RA% and SCFA concentrations compared to HC-LFOD diets. Strong positive and negative correlations were observed with faecal bacterial RA% and EIGS biomarkers (e.g., peak plasma I-FABP and CRP concentrations), and Ex-GIS, whereas no strong correlations were observed between faecal SCFA concentrations and EIGS, Ex-GIS, and performance. Several strong negative correlations were observed between several faecal bacterial groups and performance. These findings suggest that a short-term 48 h HC-HFOD diet may lead to greater changes in commensal and pathogenic bacteria RA%, which may contribute to EIGS and Ex-GIS severity, alongside negative performance outcomes. Further research with larger, more diverse cohorts is required to confirm these findings and clarify performance implications.

## Figures and Tables

**Table 1:** Shannon Equitability Index (SEI) and relative abundance (RA%) of faecal bacterial phylum-, family-, and genus-level amplicon sequence variants absolute values for baseline diet and after varied FODMAP dietary interventions.

	Baseline	HC-LFOD	HC-HFOD	*F* or *x*^2^	*p*
**Phylum**					
SEI	0.226 ± 0.061	0.212 ± 0.058	0.237 ± 0.037	*x*^2^(2) = 1.225	0.542
*Actinobacteriota*	4.595 ± 2.562	4.887 ± 2.044	5.879 ± 2.545	*F*(2,33) = 0.947	0.398
*Bacteroidota*	26.973 ± 10.405	21.965 ± 12.049	24.388 ± 9.622	*F*(2,33) = 0.653	0.527
*Firmicutes*	67.058 ± 11.501	71.815 ± 11.636	68.166 ± 8.883	*F*(2,33) = 0.644	0.532
*Proteobacteria*	0.728 ± 0.618	1.060 ± 1.176	1.243 ± 0.814	*x*^2^(2) = 3.110	0.211
**Family**					
SEI	0.251 ± 0.028	0.246 ± 0.026	0.251 ± 0.018	*F*(2,33) = 0.166	0.847
*Actinobacteriota*					
* Bifidobacteriaceae*	2.71 ± 2.44	1.894 ± 1.822	3.409 ± 2.913	*F*(2,33) = 1.251	0.300
* Coriobacteriaceae*	1.243 ± 0.932	1.483 ± 0.849	1.202 ± 0.793	*F*(2,33) = 0.362	0.699
* Eggerthellaceae*	0.441 ± 0.442	1.008 ± 1.200	0.686 ± 0.662	*t*(2) = 1.425	0.490
*Bacteroidota*					
* Bacteroidaceae*	16.338 ± 10.534	12.373 ± 9.245	10.950 ± 8.614	*F*(2,33) = 1.037	0.366
* Barnesiellaceae*	1.073 ± 1.135	0.446 ± 0.451	0.604 ± 0.787	*t*(2) = 1.748	0.417
* Prevotellaceae*	6.442 ± 9.577	7.183 ± 10.102	11.150 ± 11.239	*t*(2) = 0.400	0.819
* Rikenellaceae*	1.881 ± 1.271	1.108 ± 1.049	1.008 ± 1.015	*F*(2,33) = 2.194	0.127
* Tannerellaceae*	1.132 ± 0.625	0.828 ± 0.802	0.628 ± 0.441	*x*^2^(2) = 4.176	0.124
*Firmicutes*					
* Erysipelatoclostridiaceae*	2.114 ± 1.786	2.888 ± 2.093	2.481 ± 2.414	*x*^2^(2) = 1.257	0.533
* Erysipelotrichaceae*	0.891 ± 1.805	0.912 ± 1.975	0.439 ± 0.759	*x*^2^(2) = 0.578	0.749
* Streptococcaceae*	1.797 ± 2.393	3.271 ± 2.817	2.132 ± 1.563	*x*^2^(2) = 3.045	0.218
* Christensenellaceae*	0.934 ± 1.074	0.537 ± 0.775	0.486 ± 0.751	*x*^2^(2) = 0.918	0.632
* Lachnospiraceae*	32.072 ± 9.614	34.361 ± 7.685	33.079 ± 7.223	*x*^2^(2) = 1.311	0.519
* Butyricicoccaceae*	0.924 ± 0.489	1.146 ± 0.513	1.279 ± 0.887	*F*(2,33) = 0.901	0.416
* Oscillospiraceae*	1.772 ± 1.452	1.279 ± 1.244	1.352 ± 1.572	*x*^2^(2) = 1.218	0.544
* Ruminococcaceae*	19.164 ± 5.236	19.921 ± 7.077	17.054 ± 4.677	*F*(2,33) = 0.800	0.458
* Peptostreptococcaceae*	1.948 ± 2.032	4.191 ± 7.813	2.605 ± 3.802	*x*^2^(2) = 0.419	0.811
* Acidaminococcaceae*	0.787 ± 0.798	0.762 ± 0.842	1.243 ± 1.280	*x*^2^(2) = 0.786	0.675
* Selenomonadaceae*	2.190 ± 5.343	0.575 ± 1.747	3.822 ± 8.844	*x*^2^(2) = 0.676	0.713
* Veillonellaceae*	0.766 ± 1.125	0.832 ± 1.196	1.056 ± 1.454	*x*^2^(2) = 1.302	0.521
**Genus**					
SEI	0.277 ± 0.017	0.280 ± 0.015	0.276 ± 0.013	*F*(2,33) = 0.251	0.779
*Actinobacteriota*					
* Bifidobacterium*	2.694 ± 2.438	1.835 ± 1.808	3.395 ± 2.888	*F*(2,33) = 1.268	0.295
* Collinsella*	1.241 ± 0.973	1.483 ± 0.849	1.204 ± 0.785	*F*(2,33) = 0.350	0.707
*Bacteroidota*					
* Bacteroides*	16.298 ± 10.508	12.391 ± 9.268	10.932 ± 8.591	*F*(2,33) = 1.026	0.370
* Barnesiella*	0.920 ± 1.008	0.391 ± 0.372	0.570 ± 0.763	*x*^2^(2) = 1.335	0.513
* Prevotella*	6.130 ± 9.522	6.920 ± 9.626	10.986 ± 11.060	x^2^(2) = 0.719	0.698
* Alistipes*	1.873 ± 1.255	1.112 ± 1.046	1.006 ± 1.010	*F*(2,33) = 2.134	0.129
* Parabacteroides*	1.129 ± 0.618	0.832 ± 0.805	0.629 ± 0.440	*x*^2^(2) = 4.42	0.110
*Firmicutes*					
* Erysipelotrichaceae_UCG-003*	1.781 ± 1.806	2.528 ± 2.188	1.954 ± 2.409	*x*^2^(2) = 1.608	0.448
* Holdemanella*	0.679 ± 1.783	0.799 ± 1.970	0.354 ± 0.787	*x*^2^(2) = 0.329	0.848
* Streptococcus*	1.808 ± 2.426	3.259 ± 2.795	2.129 ± 1.561	*x*^2^(2) = 3.187	0.203
* Christensenellaceae_R-7_group*	0.923 ± 1.054	0.532 ± 0.758	0.481 ± 0.742	*x*^2^(2) = 0.809	0.667
* Agathobacter*	3.989 ± 2.909	4.571 ± 3.851	4.776 ± 4.147	*x*^2^(2) = 0.005	0.998
* Anaerostipes*	1.721 ± 1.254	1.630 ± 0.945	2.397 ± 1.905	*x*^2^(2) = 1.604	0.448
* Blautia*	8.268 ± 5.660	9.617 ± 4.137	8.139 ± 3.286	*x*^2^(2) = 1.904	0.438
* Coprococcus*	2.333 ± 1.022	2.115 ± 1.467	2.061 ± 1.592	*F*(2,33) = 0.120	0.887
* Dorea*	1.795 ± 1.041	1.972 ± 1.094	1.752 ± 1.107	*F*(2,33) = 0.121	0.887
* Fusicatenibacter*	2.495 ± 1.716	2.592 ± 1.668	2.359 ± 2.141	*x*^2^(2) = 0.592	0.744
* Lachnoclostridium*	0.411 ± 0.274	0.564 ± 0.472	1.086 ± 1.242	*x*^2^(2) = 2.658	0.265
* Lachnospira*	0.493 ± 0.472	0.665 ± 0.933	0.526 ± 0.532	*x*^2^(2) = 0.073	0.964
* Lachnospiraceae_ND3007_group*	0.760 ± 0.611	1.112 ± 0.662	0.882 ± 0.516	*x*^2^(2) = 2.307	0.316
* Lachnospiraceae_NK4A136_group*	0.657 ± 0.671	0.744 ± 0.817	0.411 ± 0.345	*x*^2^(2) = 0.657	0.720
* Roseburia*	1.016 ± 0.455	0.722 ± 0.588	0.840 ± 0.593	*F*(2,33) = 0.786	0.464
* [Eubacterium]_hallii_group*	2.673 ± 2.292	2.437 ± 0.905	2.667 ± 1.286	*x*^2^(2) = 0.637	0.727
* [Ruminococcus]_gauvreauii_group*	0.660 ± 0.590	1.075 ± 1.081	0.985 ± 0.938	*x*^2^(2) = 1.773	0.412
* [Ruminococcus]_torques_group*	1.167 ± 0.966	1.196 ± 0.746	0.971 ± 0.615	*x*^2^(2) = 0.714	0.700
* Butyricicoccus*	0.873 ± 0.509	1.063 ± 0.467	1.182 ± 0.814	*x*^2^(2) = 0.818	0.664
* UCG-002*	0.743 ± 0.791	0.488 ± 0.677	0.490 ± 0.675	*x*^2^(2) = 0.628	0.730
* Faecalibacterium*	11.079 ± 4.086	12.313 ± 4.579	11.046 ± 2.426	*F*(2,33) = 0.430	0.654
* Ruminococcus*	2.629 ± 2.209	2.662 ± 2.357	1.848 ± 1.833	*x*^2^(2) = 0.912	0.634
* Subdoligranulum*	3.358 ± 2.121	3.620 ± 2.110	2.932 ± 2.088	*F*(2,33) = 0.320	0.728
* [Eubacterium]_siraeum_group*	0.963 ± 1.577	0.471 ± 0.931	0.246 ± 0.352	*x*^2^(2) = 0.462	0.794
* Romboutsia*	1.057 ± 0.988	3.570 ± 7.852	2.080 ± 3.714	*x*^2^(2) = 0.829	0.661
* Phascolarctobacterium*	0.780 ± 0.790	0.758 ± 0.842	1.240 ± 1.275	*x*^2^(2) = 0.791	0.673
* Megamonas*	2.163 ± 5.320	0.531 ± 1.686	3.763 ± 8.787	*x*^2^(2) = 0.286	0.867
* Dialister*	0.645 ± 1.157	0.634 ± 1.127	0.546 ± 1.171	*x*^2^(2) = 0.055	0.973

Data presented as mean ± SD (*n* = 12); FODMAP, fermentable oligo-, di-, and monosaccharide and polyol; HC-LFOD, high-carbohydrate–low-FODMAP; HC-HFOD, high-carbohydrate–high-FODMAP; *F*, *F*-statistic from analysis of variance (ANOVA); *x*^2^, Chi-squared statistic; *p* < 0.05 dietary difference.

**Table 2:** Shannon Equitability Index (SEI) and relative abundance (RA%) of faecal bacterial phylum-, family-, and genus-level amplicon sequence variants relative values with change between baseline diet and high-carbohydrate–low-FODMAP (HC-LFOD) and high-carbohydrate–high-FODMAP (HC-HFOD) interventions.

Faecal Bacterial Taxonomic Levels	Baseline	HC-LFOD	∆	Test Statistic (*p*)	Baseline	HC-HFOD	∆	Test Statistic (*p*)
**Phylum**								
SEI	0.226 ± 0.061	0.212 ± 0.058	0.014 ± 0.036	1.256 (0.209) ^‡^	0.226 ± 0.061	0.237 ± 0.037	−0.011 ± 0.045	0.941 (0.347) ^‡^
*Actinobacteriota*	4.595 ± 2.562	4.887 ± 2.044	−0.293 ± 3.439	0.311 (0.792) ^†^	4.595 ± 2.562	5.879 ± 2.545	−1.284 ± 3.128	1.443 (0.177) ^†^
*Bacteroidota*	26.973 ± 10.405	21.965 ± 12.049	5.008 ± 10.037	1.733 (0.111) ^†^	26.973 ± 10.405	24.388 ± 9.622	2.585 ± 12.424	0.723 (0.485) ^†^
*Firmicutes*	67.058 ± 11.501	71.815 ± 11.636	−4.757 ± 8.936	1.840 (0.093) ^†^	67.058 ± 11.501	68.166 ± 8.883	−1.107 ± 10.261	0.371 (0.718) ^†^
*Proteobacteria*	0.728 ± 0.618	1.060 ± 1.176	−0.332 ± 1.244	0.845 (0.398) ^‡^	0.728 ± 0.618	1.243 ± 0.814	−0.515 ± 0.947	1.894 (0.085) ^†^
**Family**								
SEI	0.251 ± 0.028	0.246 ± 0.026	0.005 ± 0.023	0.696 (0.501) ^†^	0.251 ± 0.028	0.251 ± 0.018	−0.001 ± 0.020	0.100 (0.922) ^†^
*Actinobacteriota*								
* * *Bifidobacteriaceae*	2.710 ± 2.440	1.894 ± 1.822	0.864 ± 3.157	0.956 (0.360) ^†^	2.710 ± 2.440	3.409 ± 2.913	−0.703 ± 2.359	1.038 (0.322) ^†^
* * *Coriobacteriaceae*	1.243 ± 0.932	1.483 ± 0.849	−0.239 ± 0.764	1.030 (0.325) ^†^	1.243 ± 0.932	1.202 ± 0.793	0.042 ± 0.916	0.218 (0.832) ^†^
* * *Eggerthellaceae*	0.441 ± 0.442	1.008 ± 1.200	−0.567 ± 1.083	1.739 (0.082) ^‡^	0.441 ± 0.442	0.686 ± 0.662	−0.245 ± 0.529	1.782 (0.075) ^‡^
*Bacteroidota*								
* * *Bacteroidaceae*	16.338 ± 10.534	12.373 ± 9.245	3.965 ± 7.982	1.724 (0.113) ^†^	16.338 ± 10.534	10.950 ± 8.614	5.388 ± 8.332	2.244 (0.046) * ^b†^
* * *Barnesiellaceae*	1.073 ± 1.135	0.446 ± 0.451	0.627 ± 0.877	2.516 (0.029) * ^b†^	1.073 ± 1.135	0.604 ± 0.787	0.469 ± 0.580	2.705 (0.020) * ^**c**†^
* * *Prevotellaceae*	6.442 ± 9.577	7.183 ± 10.102	−0.741 ± 2.280	0.840 (0.401) ^‡^	6.442 ± 9.577	11.150 ± 11.239	−4.708 ± 6.851	2.313 (0.021) *^b‡^ 4.331 (0.001) **^c†^
* * *Rikenellaceae*	1.881 ± 1.271	1.108 ± 1.049	0.773 ± 1.036	2.566 (0.026) * ^**a**†^	1.881 ± 1.271	1.008 ± 1.015	0.873 ± 0.688	3.163 (0.009) ** ^**c**†^
* * *Tannerellaceae*	1.132 ± 0.625	0.828 ± 0.802	0.304 ± 0.795	1.607 (0.108) ^‡^	1.132 ± 0.625	0.628 ± 0.441	0.504 ± 0.564	
*Firmicutes*								0.510 (0.610) ^‡^
* * *Erysipelatoclostridiaceae*	2.114 ± 1.786	2.888 ± 2.093	−0.775 ± 1.142	2.083 (0.037) * ^**b**‡^	2.114 ± 1.786	2.481 ± 2.414	−0.368 ± 1.978	1.611 (0.107) ^‡^
* * *Erysipelotrichaceae*	0.891 ± 1.805	0.912 ± 1.975	−0.021 ± 0.210	0.000 (1.000) ^‡^	0.891 ± 1.805	0.439 ± 0.759	0.452 ± 1.201	0.785 (0.433) ^‡^
* * *Streptococcaceae*	1.797 ± 2.393	3.271 ± 2.817	−1.474 ± 3.208	1.334 (0.182) ^‡^	1.797 ± 2.393	2.132 ± 1.563	−0.335 ± 2.861	1.684 (0.092) ^‡^
* * *Christensenellaceae*	0.934 ± 1.074	0.537 ± 0.775	0.397 ± 0.930	0.892 (0.373) ^‡^	0.934 ± 1.074	0.486 ± 0.751	0.448 ± 0.847	0.235 (0.814) ^‡^
* * *Lachnospiraceae*	32.072 ± 9.614	34.361 ± 7.685	−2.289 ± 8.303	1.020 (0.308) ^‡^	32.072 ± 9.614	33.079 ± 7.223	−1.007 ± 7.358	1.421 (0.183) ^†^
* * *Butyricicoccaceae*	0.924 ± 0.489	1.146 ± 0.513	−0.222 ± 0.512	1.433 (0.180) ^†^	0.924 ± 0.489	1.279 ± 0.887	−0.355 ± 0.814	1.455 (0.146) ^‡^
* * *Oscillospiraceae*	1.772 ± 1.452	1.279 ± 1.244	0.492 ± 1.448	0.315 (0.753) ^‡^	1.772 ± 1.452	1.352 ± 1.572	0.419 ± 1.309	1.643 (0.129) ^†^
* * *Ruminococcaceae*	19.164 ± 5.236	19.921 ± 7.077	−0.757 ± 5.100	0.521 (0.613) ^†^	19.164 ± 5.236	17.054 ± 4.677	2.109 ± 4.412	0.357 (0.721) ^‡^
* * *Peptostreptococcaceae*	1.948 ± 2.032	4.191 ± 7.813	−2.242 ± 8.093	0.871 (0.384) ^‡^	1.948 ± 2.032	2.605 ± 3.802	−0.656 ± 4.498	1.224 (0.221) ^‡^
* * *Acidaminococcaceae*	0.787 ± 0.798	0.762 ± 0.842	0.025 ± 0.601	0.237 (0.813) ^‡^	0.787 ± 0.798	1.243 ± 1.280	−0.457 ± 1.183	1.095 (0.273) ^‡^
* * *Selenomonadaceae*	2.190 ± 5.343	0.575 ± 1.747	1.615 ± 3.792	1.625 (0.104) ^‡^	2.190 ± 5.343	3.822 ± 8.844	−1.632 ± 4.186	1.125 (0.261) ^‡^
* * *Veillonellaceae*	0.766 ± 1.125	0.832 ± 1.196	−0.066 ± 0.461	0.224 (0.823) ^‡^	0.766 ± 1.125	1.056 ± 1.454	−0.290 ± 0.680	
**Genus**								
SEI	0.277 ± 0.017	0.280 ± 0.015	−0.004 ± 0.016	0.749 (0.470) ^†^	0.277 ± 0.017	0.276 ± 0.013	0.000 ± 0.018	0.065 (0.949) ^†^
*Actinobacteriota*								
* * *Bifidobacterium*	2.694 ± 2.438	1.835 ± 1.808	0.859 ± 3.145	0.946 (0.364) ^†^	2.694 ± 2.438	3.395 ± 2.888	−0.700 ± 2.300	1.055 (0.314) ^†^
* * *Collinsella*	1.241 ± 0.973	1.483 ± 0.849	−0.242 ± 0.76	1.101 (0.294) ^†^	1.241 ± 0.973	1.204 ± 0.785	0.037 ± 0.915	0.140 (0.892) ^†^
*Bacteroidota*								
* * *Bacteroides*	16.298 ± 10.508	12.391 ± 9.268	3.907 ± 7.882	1.717 (0.114) ^†^	16.298 ± 10.508	10.932 ± 8.591	5.366 ± 8.321	2.234 (0.047) * ^**b**†^
* * *Barnesiella*	0.920 ± 1.008	0.391 ± 0.372	0.529 ± 0.812	2.293 (0.022) * ^**a**‡^	0.920 ± 1.008	0.570 ± 0.763	0.35 ± 0.473	2.293 (0.022) * ^**a**‡^
* * *Prevotella*	6.130 ± 9.522	6.920 ± 9.626	−0.791 ± 2.497	0.770 (0.441) ^‡^	6.130 ± 9.522	10.986 ± 11.060	−4.856 ± 6.976	1.955 (0.051) * ^**a**‡^
* * *Alistipes*	1.873 ± 1.255	1.112 ± 1.046	0.761 ± 1.012	2.605 (0.024) * ^**b**†^	1.873 ± 1.255	1.006 ± 1.010	0.866 ± 0.674	4.454 (<0.001) ** ^**c**†^
* * *Parabacteroides*	1.129 ± 0.618	0.832 ± 0.805	0.297 ± 0.784	1.647 (0.099) ^‡^	1.129 ± 0.618	0.629 ± 0.440	0.500 ± 0.557	3.112 (0.010) ** ^**c**†^
*Firmicutes*								
* * *Erysipelotrichaceae_UCG-003*	1.781 ± 1.806	2.528 ± 2.188	−0.747 ± 1.127	2.040 (0.041) * ^**a**‡^	1.781 ± 1.806	1.954 ± 2.409	−0.173 ± 1.779	0.314 (0.754) ^‡^
* * *Holdemanella*	0.679 ± 1.783	0.799 ± 1.970	−0.119 ± 0.21	1.826 (0.068) ^‡^	0.679 ± 1.783	0.354 ± 0.787	0.325 ± 1.171	0.365 (0.715) ^‡^
* * *Streptococcus*	1.808 ± 2.426	3.259 ± 2.795	−1.451 ± 3.215	1.490 (0.136) ^‡^	1.808 ± 2.426	2.129 ± 1.561	−0.321 ± 2.89	0.784 (0.433) ^‡^
* * *Christensenellaceae_R-7_group*	0.923 ± 1.054	0.532 ± 0.758	0.391 ± 0.898	0.764 (0.445) ^‡^	0.923 ± 1.054	0.481 ± 0.742	0.442 ± 0.823	1.478 (0.139) ^‡^
* * *Agathobacter*	3.989 ± 2.909	4.571 ± 3.851	−0.582 ± 3.699	0.235 (0.814) ^‡^	3.989 ± 2.909	4.776 ± 4.147	−0.788 ± 4.513	0.314 (0.754) ^‡^
* * *Anaerostipes*	1.721 ± 1.254	1.630 ± 0.945	0.092 ± 1.004	0.078 (0.937) ^‡^	1.721 ± 1.254	2.397 ± 1.905	−0.675 ± 1.354	1.490 (0.136) ^‡^
* * *Blautia*	8.268 ± 5.660	9.617 ± 4.137	−1.348 ± 3.366	1.255 (0.209) ^‡^	8.268 ± 5.660	8.139 ± 3.286	0.130 ± 4.328	0.471 (0.638) ^‡^
* * *Coprococcus*	2.333 ± 1.022	2.115 ± 1.467	0.218 ± 0.795	0.949 (0.363) ^†^	2.333 ± 1.022	2.061 ± 1.592	0.272 ± 1.087	0.867 (0.404) ^†^
* * *Dorea*	1.795 ± 1.041	1.972 ± 1.094	−0.177 ± 0.812	0.755 (0.466) ^†^	1.795 ± 1.041	1.752 ± 1.107	0.042 ± 0.889	0.165 (0.872) ^†^
* * *Fusicatenibacter*	2.495 ± 1.716	2.592 ± 1.668	−0.097 ± 1.598	0.078 (0.937) ^‡^	2.495 ± 1.716	2.359 ± 2.141	0.136 ± 1.719	0.273 (0.790) ^†^
* * *Lachnoclostridium*	0.411 ± 0.274	0.564 ± 0.472	−0.153 ± 0.369	1.437 (0.179) ^†^	0.411 ± 0.274	1.086 ± 1.242	−0.675 ± 1.124	1.961 (0.050) * ^**a**‡^
* * *Lachnospira*	0.493 ± 0.472	0.665 ± 0.933	−0.171 ± 1.065	0.157 (0.875) ^‡^	0.493 ± 0.472	0.526 ± 0.532	−0.033 ± 0.588	0.157 (0.875) ^‡^
* * *Lachnospiraceae_ND3007_group*	0.760 ± 0.611	1.112 ± 0.662	−0.352 ± 0.772	1.578 (0.143) ^†^	0.760 ± 0.611	0.882 ± 0.516	−0.122 ± 0.364	1.162 (0.270) ^†^
* * *Lachnospiraceae_NK4A136_group*	0.657 ± 0.671	0.744 ± 0.817	−0.088 ± 0.686	0.392 (0.695) ^‡^	0.657 ± 0.671	0.411 ± 0.345	0.245 ± 0.48	1.334 (0.182) ^‡^
* * *Roseburia*	1.016 ± 0.455	0.722 ± 0.588	0.294 ± 0.686	1.483 (0.166) ^†^	1.016 ± 0.455	0.840 ± 0.593	0.175 ± 0.405	1.499 (0.162) ^†^
* * *[Eubacterium]_hallii_group*	2.673 ± 2.292	2.437 ± 0.905	0.236 ± 1.712	0.314 (0.754) ^‡^	2.673 ± 2.292	2.667 ± 1.286	0.007 ± 1.871	0.471 (0.638) ^‡^
* * *[Ruminococcus]_gauvreauii_group*	0.660 ± 0.590	1.075 ± 1.081	−0.414 ± 1.074	1.412 (0.158) ^‡^	0.660 ± 0.590	0.985 ± 0.938	−0.324 ± 0.619	2.118 (0.034) * ^**a**‡^
* * *[Ruminococcus]_torques_group*	1.167 ± 0.966	1.196 ± 0.746	−0.029 ± 0.611	0.314 (0.754) ^‡^	1.167 ± 0.966	0.971 ± 0.615	0.196 ± 0.454	1.498 (0.162) ^†^
* * *Butyricicoccus*	0.873 ± 0.509	1.063 ± 0.467	−0.189 ± 0.497	1.317 (0.215) ^†^	0.873 ± 0.509	1.182 ± 0.814	−0.308 ± 0.746	1.255 (0.209) ^‡^
* * *UCG-002*	0.743 ± 0.791	0.488 ± 0.677	0.256 ± 0.739	0.800 (0.424) ^‡^	0.743 ± 0.791	0.490 ± 0.675	0.253 ± 0.584	1.172 (0.241) ^‡^
* * *Faecalibacterium*	11.079 ± 4.086	12.313 ± 4.579	−1.234 ± 5.575	0.767 (0.459) ^†^	11.079 ± 4.086	11.046 ± 2.426	0.033 ± 4.014	0.028 (0.978) ^†^
* * *Ruminococcus*	2.629 ± 2.209	2.662 ± 2.357	−0.033 ± 2.272	0.051 (0.960) ^†^	2.629 ± 2.209	1.848 ± 1.833	0.781 ± 1.716	1.376 (0.169) ^‡^
* * *Subdoligranulum*	3.358 ± 2.121	3.620 ± 2.110	−0.262 ± 1.527	0.626 (0.544) ^†^	3.358 ± 2.121	2.932 ± 2.088	0.426 ± 1.288	1.105 (0.293) ^†^
* * *[Eubacterium]_siraeum_group*	0.963 ± 1.577	0.471 ± 0.931	0.491 ± 0.825	1.963 (0.050) * ^**a**‡^	0.963 ± 1.577	0.246 ± 0.352	0.717 ± 1.406	1.947 (0.050) * ^**a**‡^
* * *Romboutsia*	1.057 ± 0.988	3.570 ± 7.852	−2.513 ± 7.959	1.783 (0.075) ^‡^	1.057 ± 0.988	2.080 ± 3.714	−1.023 ± 4.145	0.356 (0.722) ^‡^
* * *Phascolarctobacterium*	0.780 ± 0.790	0.758 ± 0.842	0.023 ± 0.589	0.178 (0.858) ^‡^	0.780 ± 0.790	1.240 ± 1.275	−0.460 ± 1.174	1.173 (0.241) ^‡^
* * *Megamonas*	2.163 ± 5.320	0.531 ± 1.686	1.632 ± 3.824	1.604 (0.109) ^‡^	2.163 ± 5.320	3.763 ± 8.787	−1.600 ± 4.115	1.069 (0.285) ^‡^
* * *Dialister*	0.645 ± 1.157	0.634 ± 1.127	0.01 ± 0.537	0.000 (1.000) ^‡^	0.645 ± 1.157	0.546 ± 1.171	0.099 ± 0.437	0.272 (0.785) ^‡^

Data presented as mean ± SD (*n* = 12): ** *p* < 0.01; * *p* < 0.05 vs. baseline. Figures in bold represent significance *p* < 0.05. ^a^ Small effects (Cohens *d* < 0.2–0.49). ^b^ Medium effects (Cohens *d* = 0.5–0.79). ^c^ Large effects (Cohens *d* ≥ 0.8). ^†^ *t*-test. ^‡^ Wilcoxon test.

**Table 3:** Correlations between faecal bacterial Shannon Equitability Index (SEI) and relative abundance (RA%) and exercise-induced gastrointestinal syndrome biomarkers, exercise-associated gastrointestinal symptoms (Ex-GIS), and performance.

Faecal Bacterial Taxonomic Levels	I-FABP	sCD14	CRP	OCTT	Ex-GIS	Performance
**Phylum**						
SEI	0.221 ^‡^	−0.018 ^‡^	−0.093 ^‡^	−0.459 *^a^ ^‡^	−0.118 ^‡^	−0.035 ^‡^
*Actinobacteriota*	0.268 ^†^	0.257 ^‡^	0.159 ^†^	−0.178 ^†^	0.084 ^†^	−0.429 *^a^ ^†^
*Bacteroidota*	0.248 ^†^	−0.155 ^‡^	−0.131 ^†^	−0.267 ^†^	−0.079 ^†^	0.096 ^†^
*Firmicutes*	−0.269 ^†^	0.127 ^‡^	0.105 ^†^	0.336 ^†^	0.068 ^†^	0.010 ^†^
*Proteobacteria*	−0.480 *^a^ ^‡^	0.087 ^‡^	0.589 **^a^ ^‡^	−0.080 ^‡^	−0.008 ^‡^	−0.294 ^‡^
**Family**						
SEI	0.248 ^†^	0.131 ^†^	0.200 ^†^	−0.300 ^†^	−0.087 ^†^	−0.039 ^†^
*Actinobacteriota*						
* Bifidobacteriaceae*	0.196 ^‡^	0.216 ^‡^	0.114 ^‡^	−0.036 ^‡^	0.247 ^‡^	−0.318 ^‡^
* Coriobacteriaceae*	−0.309 ^†^	0.041 ^‡^	0.379 ^†^	0.266 ^†^	−0.043 ^‡^	−0.647 ** ^a†^
* Eggerthellaceae*	0.305 ^‡^	−0.200 ^‡^	−0.211 ^‡^	−0.097 ^‡^	−0.196 ^‡^	0.066 ^‡^
*Bacteroidota*						
* Bacteroidaceae*	0.657 ** ^b†^	0.203 ^‡^	−0.233 ^†^	0.034 ^†^	0.286 ^‡^	0.218 ^†^
* Barnesiellaceae*	0.287 ^‡^	0.080 ^‡^	0.284 ^‡^	−0.320 ^‡^	0.476 * ^a‡^	0.097 ^‡^
* Prevotellaceae*	−0.351 ^‡^	−0.307 ^‡^	−0.056 ^‡^	−0.249 ^‡^	−0.291 ^‡^	0.070 ^‡^
* Rikenellaceae*	0.602 ** ^b‡^	0.367 ^‡^	0.215 ^‡^	−0.114 ^‡^	0.603 ** ^b‡^	−0.045 ^‡^
* Tannerellaceae*	0.373 ^‡^	0.206 ^‡^	−0.070 ^‡^	−0.060 ^‡^	0.304 ^‡^	−0.157 ^‡^
*Firmicutes*						
* Erysipelatoclostridiaceae*	−0.336 ^‡^	0.093 ^‡^	0.050 ^‡^	0.171 ^‡^	−0.381 ^‡^	−0.325 ^‡^
* Erysipelotrichaceae*	0.135 ^‡^	0.104 ^‡^	0.071 ^‡^	0.120 ^‡^	0.017 ^‡^	0.194 ^‡^
* Streptococcaceae*	0.107 ^‡^	0.140 ^‡^	−0.418 ^‡^	0.248 ^‡^	−0.373 ^‡^	0.179 ^‡^
* Christensenellaceae*	0.515 ** ^a‡^	0.135 ^‡^	0.287 ^‡^	−0.155 ^‡^	0.440 * ^a‡^	0.050 ^‡^
* Lachnospiraceae*	−0.256 ^†^	0.242 ^‡^	0.056 ^†^	0.440 * ^a†^	0.112 ^‡^	−0.222 ^†^
* Butyricicoccaceae*	−0.194 ^†^	−0.189 ^‡^	0.076 ^†^	−0.157 ^†^	−0.579 ** ^a‡^	0.004 ^†^
* Oscillospiraceae*	0.496 * ^a‡^	0.103 ^‡^	0.274 ^‡^	−0.215 ^‡^	0.428 * ^a‡^	0.114 ^‡^
* Ruminococcaceae*	0.192 ^†^	0.272 ^‡^	−0.028 ^†^	−0.186 ^†^	0.302 ^‡^	−0.399 ^†^
* Peptostreptococcaceae*	0.113 ^‡^	0.220 ^‡^	−0.023 ^‡^	0.047 ^‡^	0.062 ^‡^	0.140 ^‡^
* Acidaminococcaceae*	−0.164 ^‡^	−0.167 ^‡^	0.525 * ^a‡^	−0.544 ** ^a‡^	0.239 ^‡^	0.208 ^‡^
* Selenomonadaceae*	−0.242 ^‡^	−0.346 ^‡^	0.281 ^‡^	0.064 ^‡^	−0.204 ^‡^	0.398 ^‡^
* Veillonellaceae*	0.306 ^‡^	0.023 ^‡^	−0.418 ^‡^	0.129 ^‡^	−0.215 ^‡^	−0.044 ^‡^
**Genus**						
SEI	−0.107 ^‡^	0.428 * ^a‡^	0.199 ^‡^	0.208 ^‡^	0.369 ^‡^	−0.543 ** ^a‡^
*Actinobacteriota*						
* Bifidobacterium*	0.196 ^‡^	0.216^‡^	0.114 ^‡^	−0.036 ^‡^	0.247 ^‡^	−0.318 ^‡^
* Collinsella*	−0.301 ^†^	0.030^‡^	0.366 ^†^	0.273 ^†^	−0.053 ^‡^	−0.638 ** ^b†^
*Bacteroidota*						
* Bacteroides*	0.661 ** ^b†^	0.192 ^‡^	−0.240 ^†^	0.034 ^†^	0.285 ^‡^	0.221 ^†^
* Barnesiella*	0.289 ^‡^	0.079 ^‡^	0.297 ^‡^	−0.320 ^‡^	0.486 * ^a‡^	0.100 ^‡^
* Prevotella*	−0.336 ^‡^	−0.299 ^‡^	−0.053 ^‡^	−0.276 ^‡^	−0.284 ^‡^	0.070 ^‡^
* Alistipes*	0.602 ** ^b‡^	0.367 ^‡^	0.215 ^‡^	−0.114 ^‡^	0.603 ** ^a‡^	−0.045 ^‡^
* Parabacteroides*	0.373 ^‡^	0.194 ^‡^	−0.076 ^‡^	−0.050 ^‡^	0.301 ^‡^	−0.147 ^‡^
*Firmicutes*						
* Erysipelotrichaceae_UCG-003*	−0.383 ^‡^	−0.135 ^‡^	−0.175 ^‡^	0.431 * ^a‡^	−0.251 ^‡^	−0.286 ^‡^
* Holdemanella*	0.022 ^‡^	−0.110 ^‡^	0.154 ^‡^	0.258 ^‡^	0.020 ^‡^	0.148 ^‡^
* Streptococcus*	0.107 ^‡^	0.140 ^‡^	−0.418 ^‡^	0.248 ^‡^	−0.373 ^‡^	0.179 ^‡^
* Christensenellaceae_R-7_group*	0.515 **^a^ ^‡^	0.135 ^‡^	0.287 ^‡^	−0.155 ^‡^	0.440 * ^a‡^	0.050 ^‡^
* Agathobacter*	−0.133 ^‡^	0.316 ^‡^	0.495 * ^a‡^	0.099 ^‡^	0.365 ^‡^	−0.751 ** ^b‡^
* Anaerostipes*	−0.311 ^†^	0.048 ^‡^	0.129 ^†^	0.214 ^†^	−0.335 ^‡^	0.211 ^†^
* Blautia*	0.093 ^†^	0.055 ^‡^	−0.207 ^†^	0.404 ^†^	−0.078 ^‡^	0.288 ^†^
* Coprococcus*	0.497 * ^a‡^	0.012 ^‡^	−0.004 ^‡^	−0.004 ^‡^	0.427 *^a^ ^‡^	0.338 ^‡^
* Dorea*	−0.333 ^‡^	−0.176 ^‡^	−0.286 ^‡^	0.127 ^‡^	−0.614 ** ^b‡^	0.075 ^‡^
* Fusicatenibacter*	−0.013 ^‡^	0.432 * ^a‡^	−0.136 ^‡^	0.346 ^‡^	0.137 ^‡^	−0.654 ** ^b‡^
* Lachnoclostridium*	−0.063 ^‡^	−0.237 ^‡^	0.100 ^‡^	−0.156 ^‡^	0.138 ^‡^	−0.416 * ^a‡^
* Lachnospira*	0.250 ^‡^	0.505 * ^a‡^	−0.085 ^‡^	0.020 ^‡^	0.240 ^‡^	−0.142 ^‡^
* Lachnospiraceae_ND3007_group*	−0.503 ^†^	0.026 ^‡^	0.143 ^†^	−0.101 ^†^	0.029 ^‡^	−0.424 * ^a‡^
* Lachnospiraceae_NK4A136_group*	0.334 ^‡^	0.029 ^‡^	0.287 ^‡^	0.097 ^‡^	0.314 ^‡^	0.050 ^‡^
* Roseburia*	0.423 * ^a‡^	0.050 ^‡^	−0.231 ^‡^	0.154 ^‡^	−0.043 ^‡^	0.297 ^‡^
* [Eubacterium]_hallii_group*	−0.245 ^†^	−0.200 ^‡^	0.120 ^†^	0.392 ^†^	−0.060 ^‡^	0.100 ^†^
* [Ruminococcus]_gauvreauii_group*	−0.089 ^‡^	0.043 ^‡^	−0.656 ** ^a‡^	0.397 ^‡^	−0.330 ^‡^	0.161 ^‡^
* [Ruminococcus]_torques_group*	−0.301 ^‡^	−0.241 ^‡^	−0.359 ^‡^	0.038 ^‡^	−0.059 ^‡^	0.193 ^‡^
* Butyricicoccus*	−0.450 * ^a‡^	−0.220 ^‡^	−0.197 ^‡^	−0.016 ^‡^	0.530 ** ^a‡^	−0.053 ^‡^
* UCG-002*	−0.508 * ^a‡^	0.201 ^‡^	0.277 ^‡^	−0.159 ^‡^	0.452 * ^a‡^	0.077 ^‡^
* Faecalibacterium*	0.144 ^†^	0.270 ^‡^	−0.193 ^†^	−0.182 ^†^	−0.049 ^‡^	−0.448 * ^a‡^
* Ruminococcus*	0.206 ^‡^	0.305 ^‡^	0.300 ^‡^	−0.358 ^‡^	0.532 ** ^a‡^	−0.159 ^‡^
* Subdoligranulum*	0.297 ^†^	0.184 ^‡^	0.190 ^†^	−0.023 ^†^	0.378 ^‡^	0.024 ^†^
* [Eubacterium]_siraeum_group*	0.092 ^‡^	0.207 ^‡^	−0.107 ^‡^	−0.159 ^‡^	0.330 ^‡^	−0.066 ^‡^
* Romboutsia*	0.060 ^‡^	0.251 ^‡^	−0.033 ^‡^	0.123 ^‡^	0.078 ^‡^	0.265 ^‡^
* Phascolarctobacterium*	−0.162 ^‡^	−0.173 ^‡^	0.515 ** ^a‡^	0.544 ** ^a‡^	0.253 ^‡^	0.213 ^‡^
* Megamonas*	−0.607 ** ^b‡^	−0.407 * ^a‡^	0.053 ^‡^	−0.054 ^‡^	−0.388 ^‡^	0.231 ^‡^
* Dialister*	0.306 ^‡^	−0.041 ^‡^	−0.440 ^‡^	0.281 ^‡^	−0.302 ^‡^	−0.161 ^‡^

I-FABP, plasma intestinal fatty acid binding protein pre- to peakpost-exercise magnitude of change; sCD14, plasma soluble cluster of differentiation 14; CRP, plasma C-reactive protein concentrations; OCTT, orocaecal transit time. ^a^ Moderate correlation (0.400–0.599). ^b^ Strong correlation (≥0.600). ^†^ Pearson’s *r*. ^‡^ Spearman’s rho (*r_s_*). ** *p* < 0.01 and * *p* < 0.05. Figures in bold represent significance *p* < 0.05.

## Data Availability

The data presented in this study are available upon request from the corresponding author, in accordance with the ethics committee approval procedures, due to privacy and confidentiality concerns.

## Abbreviations

The following abbreviations are used in this manuscript:

ASV	Amplicon sequence variant
CI	Confidence interval
CH_4_	Methane
CHO	Carbohydrate
CO_2_	Carbon dioxide
CRP	C-reactive protein
CV	Coefficient of variation
DNA	Deoxyribonucleic acid
DNAse	Deoxyribonuclease
Ex-GIS	Exercise-associated gastrointestinal symptoms
EIGS	Exercise-induced gastrointestinal syndrome
ELISA	Enzyme-linked immunosorbent assay
FODMAP	Fermentable oligo-, di-, and monosaccharide and polyol
GIS	Gastrointestinal symptoms
H_2_	Hydrogen
HC-HFOD	High-carbohydrate–high FODMAP
HC-LFOD	High-carbohydrate–low FODMAP
IBS	Irritable bowel syndrome
I-FABP	Intestinal fatty acid binding protein
ISTDs	Internal standards
IU	International units
km	Kilometre
LC-MS	Liquid chromatography–mass spectrometry
MBCA	Medical body composition analyser
MeOH	Methanol
MEOTime	Mean effect over time
mVAS	Modified visual analogue scale
OCTT	Orocaecal transit time
PCR	Polymerase chain reaction
QC	Quality control
rRNA	Ribosomal RNA
RA%	Relative abundance percent
RH	Relative humidity
RNAse	Ribonuclease
sCD14	Soluble cluster of differentiation 14
SCFAs	Short-chain fatty acids
SEI	Shannon Equitability Index
SEM	Standard error of the mean
SD	Standard deviation
UK	United Kingdom
USA	United States of America
